# Impact of Environmental Conditions on the Survival of *Cryptosporidium* and *Giardia* on Environmental Surfaces

**DOI:** 10.1155/2014/210385

**Published:** 2014-06-17

**Authors:** Absar Alum, Isra M. Absar, Hamas Asaad, Joseph R. Rubino, M. Khalid Ijaz

**Affiliations:** ^1^Arizona State University, Tempe, AZ 85287, USA; ^2^BioDetek, 1815 West First Avenue, Suite 125, Mesa, AZ 85202, USA; ^3^RB, One Philips Parkway, Montvale, NJ 07645, USA

## Abstract

The objective of this study was to find out the impact of environmental conditions on the survival of intestinal parasites on environmental surfaces commonly implicated in the transmission of these parasites. The study was performed by incubating *Cryptosporidium* and *Giardia* (oo)cysts on environmentally relevant surfaces such as brushed stainless steel, formica, ceramic, fabric, and skin. Parallel experiments were conducted using clean and soiled coupons incubated under three temperatures. The die-off coefficient rates (*K*) were calculated using first-order exponential formula. For both parasites, the fastest die-off was recorded on fabric, followed by ceramic, formica, skin, and steel. Die-off rates were directly correlated to the incubation temperatures and surface porosity. The presence of organic matter enhanced the survivability of the resting stages of test parasites. The decay rates calculated in this study can be used in models for public health decision-making process and highlights the mitigation role of hand hygiene agents in their prevention and control.

## 1. Introduction 

Populations in different parts of the world face diverse parasitic challenges. The public health managers around the world face different challenges because the diversity of parasites endemic in an area is influenced by a variety of factors [1–4]. For example,* Enterobius vermicularis* is more prevalent in temperate areas [5], and incidence of* Ascaris lumbricoides* is more common in tropical regions [6].

To reduce the infectious disease load in communities, public health managers have a variety of intervention tools; however, the effectiveness of these tools is impacted by environmental, cultural, and socioeconomic conditions. For example, hand washing can be effective under a broad variety of conditions [7–9]. However, the effectiveness of these interventions is impacted by personal and cultural norms [10]. The role of hand washing as an intervention for improving public health safety is well established. However, it is practiced significantly differently across societies and cultures. For example, in Peru, only 11% of people wash their hands after defecation and the use of soap is still more rare [11]. In a recent survey, we found that 14% of children and adults in India and 7% in Bangladesh carry the neglected enteric parasites on their hands [12]. Furthermore, these parasites have been implicated in impacting both the physical and the cognitive development of children in the affected regions of the world highlighting the mitigational role of hygiene [13]. In addition to personal hygiene practices, environmental factors also influence the richness of parasite species and intensity of infection in the host species [14] by impacting the survival of the environmental stages of these intestinal parasites. Among environmental conditions, temperature and relative humidity are known to have significant effect on the survival of (oo)cysts. Temperature changes have been associated with the parasite development rates [15, 16], while moisture and relative humidity have been reported for maintaining parasite pressure [17, 18]. Optimum moisture and temperature conditions are critical for maintaining the vitality of food stored in (oo)cysts until they are ready for the next stage in their life cycle [19]. Given the importance of temperature and relative humidity in parasite life cycle, it is imperative to study the impact of these factors on intestinal parasites that have been neglected in the scientific studies during the recent years [7].

Environmental, cultural, and socioeconomic conditions factors along with nature of public health threat such as pathogen survival are important inputs in public health decision-making processes. Reliability of any model is tied to the accuracy of data used in the model. Information on the survival of intestinal parasites and public health relevant conditions is sparse and scattered in the literature. There is a need for comprehensive study to generate scientific data to facilitate decision-making process for addressing the public health threat from intestinal parasites.

In this study, survival of* Cryptosporidium* and* Giardia* (oo)cysts on animate and inanimate surfaces was investigated under environmental conditions of public health relevance.

## 2. Methods

### 2.1. Parasites

*C. parvum* oocysts (Iowa isolate) were obtained from the Sterling Parasitology Laboratory, the University of Arizona, Tucson, AZ, USA. The* Giardia muris* cysts were obtained from Dr. Shivaji Ramalingam (Oregon Health Sciences University, Portland, OR, USA). Upon receipt, all the stocks of (oo)cysts were stored at 4°C until being used for assays. Concentrations of all the stocks and working solutions were determined by direct count using a hemocytometer.

### 2.2. Coupons for Survival Studies

Five types of materials representing animate and inanimate prototype surfaces of different porosity and surface roughness were selected for the study. The materials included brushed stainless steel 18 Ga (MetalsDepot, Winchester, KY, USA), formica (Home Depot, Gilbert, AZ, USA), ceramic plates (Home Depot, Gilbert, AZ, USA), fabric (100% cotton) (Testfabrics, West Pittson, PA, USA), and synthetic skin (Bioscience, Castro Valley, CA, USA). For each material, coupons measuring 1 square inch (2.5 cm × 2.5 cm) were used in this study. Porosity of the selected material was determined using water saturation method [20].

### 2.3. Experimental Conditions

Survival of* Cryptosporidium* and* Giardia* (oo)cysts was studied on brushed stainless steel, formica, ceramic, fabric, and synthetic skin. For each test parasite, (oo)cysts were inoculated on each type of material with and without organic matter—10 mg/0.2 mL bovine serum albumen (Mallinckrodt, Paris, KY, USA). The inoculated coupons were incubated at 15°C/50% RH, 25°C/50% RH, and 37°C/70% RH for a specified time period. All the experiments were performed in triplicate. After the specified incubation period, inoculated coupons were processed to study the viability/infectivity of each parasite.

### 2.4. Environmental Chamber

Inoculated coupons were placed in glass jars that were set up according to ASTM Standard E96. The relative humidity inside these jars was maintained by using anhydrous calcium chloride as a desiccant. These coupon holding jars were placed in trays inside environmental chambers that were built according to the ASTM method D3273 using polypropylene tanks. Digital thermometer/hygrometers were installed in environmental chambers to constantly monitor the relative humidity and temperature.

### 2.5. Viability and Infectivity Studies

The viability of* Cryptosporidium* oocysts and* Giardia* cysts was determined using methods described previously [21, 22]. Sample processing and PCR conditions were based on the procedure previously described [22].

### 2.6. Calculation of Die-Off Rates

The first-order exponential formula was used to simulate (oo)cysts die-off on five surfaces at different environmental conditions. The equation is as follows:

(1) $$\begin{array}{c} y_{t}\left(t\right)=y_{0}e^{-Kt}, \end{array}$$

where *K* is the die-off rate coefficient and *y*_0_ and *y*_*t*_ are the numbers of (oo)cysts at time zero, under initial conditions and at time *t*, respectively. If normalized by the initial numbers of (oo)cysts, (1) can be rewritten as follows:

(2) $$\begin{array}{c} y_{t}^{\prime }\left(t\right)=e^{-Kt}\;\text{or}\;\mathrm{In}y_{t}^{\prime }\left(t\right)=-Kt, \end{array}$$

where

(3) $$\begin{array}{c} y_{t}^{\prime }\left(t\right)=\frac{y_{t}\left(t\right)}{y_{0}}. \end{array}$$

In (2), *K* is independent of the initial numbers of parasitic (oo)cysts and represents a constant die-off rate over the entire incubation period.

## 3. Results 

**Figure 1:**
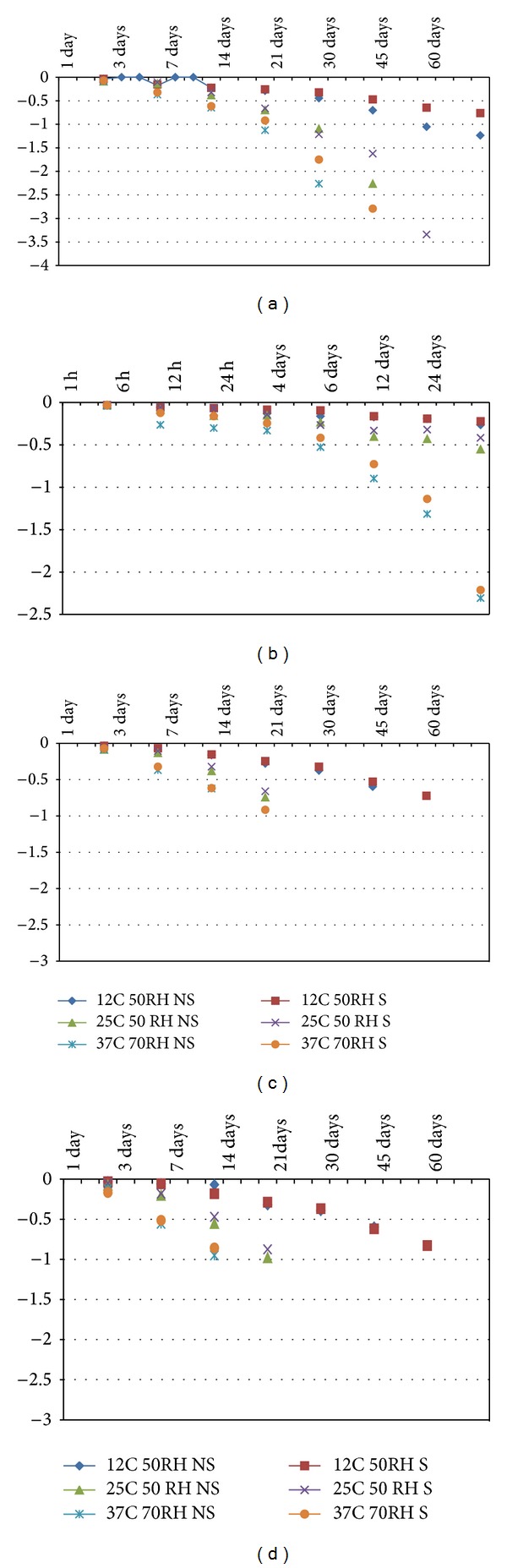
*K* values for the first-order die-off rate of* Giardia* on (a) stainless steel, (b) skin, (c) formica, and (d) fabric.

**Figure 2:**
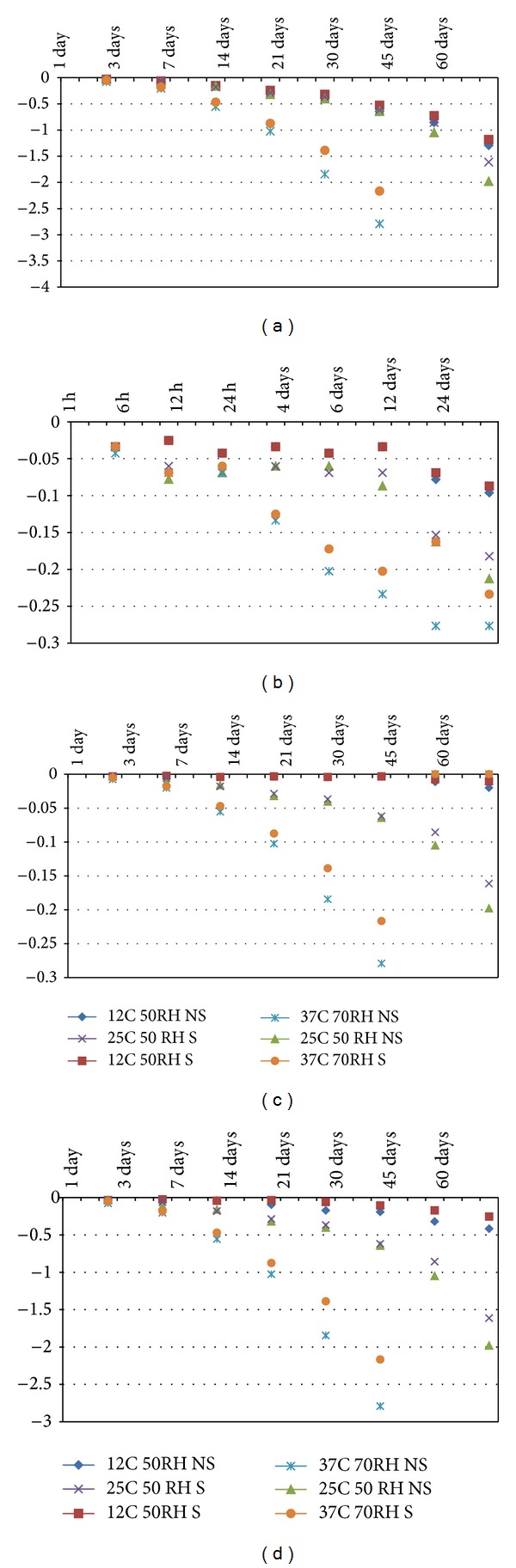
*K* values for the first-order die-off rate of* Cryptosporidium* on (a) stainless steel, (b) skin, (c) formica, and (d) fabric.

The survival/infectivity of* Cryptosporidium* oocysts and* Giardia* cysts was studied under different environmental conditions, and inactivation kinetics are presented here. In general, parasites survival was inversely correlated with the storage temperature and porosity of the surface. The temporal decay of* Cryptosporidium* oocysts and* Giardia *cysts under various environmental conditions (temperature, relative humidity, and organic material) and on different surfaces (microlevel variation in porosity and roughness) is presented in Figures 1 and 2, and the results are summarized in Table 1. On clean (nonsoiled) surfaces stored at 25°C, the die-off rate of* Giardia *cysts ranged from −0.25754 to −0.7764, whereas, for similar samples stored at 12°C and 37°C, the cysts die-off rate ranged from −0.14704 to −0.34612 and −0.36001 to −0.89851, respectively. Similarly, under various test conditions, the overall trends of the* Cryptosporidium* oocysts die-off were similar to the one of* Giardia *cysts; however, the level of die-off for both parasites was different under comparable conditions. The* Cryptosporidium* oocysts die-off on clean surfaces incubated at 37°C ranged from −0.16308 to −1.08403, whereas incubation at 12°C and 25°C resulted in oocysts die-off rates ranging from −0.04837 to −0.46996 and −0.09552 to −0.58958, respectively. Presence of organic matter on the surface had a protective effect on the survival of both parasites; however, this protective effect was more consistent for* Giardia *cysts than* Cryptosporidium* oocysts (Table 1). The results of this study concur with previous studies reporting the importance of environmental conditions such as temperature on the survival and transmission of intestinal parasites [23–25].

## 4. Discussion

In surface water, oocysts and cysts can survive for months [26–29]. Under natural conditions, the die-off rate of* Cryptosporidium* oocysts in water is 0.005–0.037 log10-units per day. For* Giardia*, the die-off rate is higher and more temperature-dependent, varying between 0.015 log10-units per day at 1°C and 0.28 log10-units per day at 23°C [27]. However, when present on surfaces or in solids (soil or sludge), different parasites may respond differently to variations in environmental conditions such as temperature, relative humidity (RH), porosity, and organic matter. For example, under conditions of severe desiccation,* Eimeria* remains viable for a significantly longer period of time than the* Cryptosporidium* oocysts [30]. After reviewing the available data on the relation of die-off of different parasites to moisture and temperature, the USEPA concluded that no single microorganism is really adequate to represent all other parasites [31].

### 4.1. Effect of Temperature

Over the course of the study, higher temperature resulted in increased die-off of* Cryptosporidium* and* Giardia*. Temperature has been regarded as one of the most critical factors in the (oo)cysts survival in the environment [29, 31–33]. In saturated soil at 37°C, 1-log reduction in the viability of* Cryptosporidium* oocysts was recorded in 10 days, whereas storage of oocysts for months under similar conditions at 15°C did not result in the loss of viability [34, 35]. In a study, 10^6^ oocysts of* Cryptosporidium* oocysts were stored for 10 days in dry soils at 32°C, and no loss of viable oocysts was recorded by PCR-based assay [35]. Similar survival trends have been reported by others, reporting complete die-off in 24 weeks, 8 weeks, and 72 hours for 15°C, 25°C, and 37°C, respectively [35, 36]. The higher inactivation reported by these researchers may be due to diurnal changes in temperature which can result in the rapid breakdown of energy reserves, which are vital for viability and infectivity of resting stages of parasites [35, 36]. In addition, in the presence of bacterial extracellular enzymes, in just 7 hours, 1-log reduction in the number of viable/infectious* Cryptosporidium* oocysts has been reported [34]. These findings are critical when comparing the results of studies conducted in clean environment and under field conditions.

Various estimates have been made for rate of inactivation of parasite (oo)cysts. These resting forms of enteric parasites are more resistant to environmental conditions than the nonencysted forms. The survival of (oo)cysts depends on the type of parasites and the conditions to which they are exposed. Defining a quantified relationship between the die-off rate and environmental stresses for enteric parasite-contaminated environmental surfaces has received little attention in the literature. The USEPA has formulated default die-off values applicable to all kinds of (oo)cysts for different temperatures and incubation conditions [37]. The die-off rates of different parasites reported in this study for 15°C, 25°C, and 37°C are supported by results of previous studies. O'Donnell et al. reported die-off rates of 10^(−0.2)^ and 10^(−0.3)^ per month for* Ascaris* ova in aerobically treated sludge and anaerobic treated sludge, respectively [38]. For* Giardia,* die-off rates of 0.029 log10 day^−1^ and 0.37 log10 day^−1^ have been reported for water and sediment, respectively [39]. For* Cryptosporidium* oocysts, kept in water, soils, and feces? Needs to clarify *K* values of −0.041 and −0.130 per day have been reported for 15°C and 25°C, respectively [40]. In the present study, similar die-off *K* values were estimated for* Cryptosporidium* oocysts deposited on formica for one week.

### 4.2. Surface Porosity and Roughness

Survival of some common parasite (oo)cysts in solids (sand, loam, and sludge) of different porosity has been studied [34, 37]; however, no information is available on the survival of the (oo)cysts of the neglected intestinal parasites such as* Cryptosporidium and Giardia lamblia* on animate and inanimate surfaces. In general, greater viability loss was noted for all test enteric parasitic (oo)cysts incubated on fabric and ceramic compared to steel and formica. This difference in viability of various types of surfaces appears to be inversely related to the surface porosity. It can be argued that parasites (oo)cysts on higher porosity surfaces may be exposed to increased osmotic stress, which can be a factor for higher die-off rate on such surfaces. It is known that desiccation can cause lethal impact on oocysts and they can lose viability after being subjected to stress driven by the interaction of water contents and texture of storage material [38, 41]. Nasser et al. found that incubation for 10 days in dry loamy soil at 32°C resulted in a 2.5-log reduction in oocysts by cell culture assay but no such decrease was recorded by PCR assay [34]. However, only 1-log reduction was recorded in saturated soil at similar temperature. They also demonstrated that the viability of oocysts in saturated loamy soil at 15°C remained unchanged for months. Jenkins et al. reported that estimated *K* values for soil at 25°C were increased from 0.014 to 0.416 day^−1^ when the soil water potential was decreased from −0.10 to −3.2 MPa [42]. Therefore, the direct correlation between higher die-off rates of parasites (oo)cysts and higher porosity of surfaces noted in this study is supported by previous reports.

### 4.3. Organic Load

Presence of organic matter and soil load is known to impact the survival of parasites. In the present study, presence of organic matter generally enhanced the survival of parasites (oo)cysts. Based on the extensive amount of parasite survival data, USEPA has calculated generic die-off values of 0.000533/hour, 0.00041/hour, and 0.7845/hour for* Ascaris* ova in moist soil, on soil surface, and on plant surface, respectively [37]. It has been shown that soil particle or organic matter provides protection to parasites against environmental factors affecting their viability. This supports the finding of the present study reporting better survival of all parasites (oo)cysts when studied under soiled conditions.

Due to experimental and logistic limitations, most of the studies investigating parasite survival under different environmental conditions do not use factorial experimental design. Therefore, data from such studies cannot be used to isolate the impact of temperature from other variables on the survival of parasite (oo)cysts. This is the first study that used a factorial experimental design to investigate the impact of temperature, relative humidity, organic load, and surface type on the survival of parasites' resting stages [(oo)cysts] of parasites. Although with the present knowledge it is not possible to precisely define the role of environmental variables (temperature, RH, porosity, organic matter, the nutrient concentration, and the parasitic/microbial communities) in the survival of parasites (oo)cysts, recovery of neglected enteric parasitic (oo)cysts/ova from naturally contaminated hands of children reported previously [11] coupled with the results of the present study provide valuable information for predicting their survival under variable conditions of public health significance and help in designing hygienic measures for interruption of their dissemination to both animal and human populations.

## Figures and Tables

**Table 1:** Summary of die-off rates (the first-order *K* values) of *Giardia* cysts and *Cryptosporidium* oocysts on a variety of surfaces incubated in different environmental conditions.

Parasite	Test material	Test conditions					
		12°C 50% RH		25°C 50% RH		37°C 70% RH	
		NS	S	NS	S	NS	S
*Giardia *	Fabric	−0.24609	−0.3409	−0.45487	−0.39509	−0.54514	−0.5132
	Ceramic	−0.2572	−0.29622	−0.35363	−0.28729	−0.36001	−0.47733
	Formica	−0.2572	−0.29622	−0.33656	−0.28729	−0.36001	−0.48086
	Skin	−0.14704	−0.11569	−0.25754	−0.20598	−0.74951	−0.6331
	Stainless steel	−0.34612	−0.35842	−0.7764	−1.04699	−0.89851	−1.07793
	Average *K*	**−0.25073**	**−0.28149**	**−0.4358**	**−0.44453**	**−0.58264**	**−0.63648**

*Cryptosporidium *	Fabric	−0.16294	−0.09118	−0.58958	−0.5037	−1.08403	−0.85624
	Ceramic	−0.46996	−0.40785	−0.58958	−0.5037	−1.08403	−0.85624
	Formica	−0.0663	−0.04954	−0.58958	−0.5037	−0.81303	−0.64218
	Skin	−0.04837	−0.04609	−0.09552	−0.08723	−0.16308	−0.13251
	Stainless steel	−0.46996	−0.40785	−0.58958	−0.5037	−1.08403	−0.85624
	Average *K*	**−0.24351**	**−0.2005**	**−0.49077**	**−0.42041**	**−0.84564**	**−0.66868**

S: surface soiled with 5% organic matter; NS: surface not soiled (clean).
